# Camouflage using three-dimensional surface disruption

**DOI:** 10.1098/rsbl.2022.0596

**Published:** 2023-08-02

**Authors:** Jemma King, Jan M. Hemmi, Jennifer L. Kelley

**Affiliations:** ^1^ School of Biological Sciences, The University of Western Australia, 35 Stirling Highway, Crawley 6009, Perth, Australia; ^2^ UWA Oceans Institute, The University of Western Australia, 35 Stirling Highway, Crawley 6009, Perth, Australia

**Keywords:** disruptive coloration, background matching, crypsis, predation, perception, vision

## Abstract

Disruptive markings are common in animal patterns and can provide camouflage benefits by concealing the body's true edges and/or by breaking the surface of the body into multiple depth planes. Disruptive patterns that are accentuated by high contrast borders are most likely to provide false depth cues to enhance camouflage, but studies to date have used visual detection models or humans as predators. We presented three-dimensional-printed moth-like targets to wild bird predators to determine whether: (1) three-dimensional prey with disrupted body surfaces have higher survival than three-dimensional prey with continuous surfaces, (2) two-dimensional prey with disruptive patterns or enhanced edge markings have higher survival than non-patterned two-dimensional prey. We found a survival benefit for three-dimensional prey with disrupted surfaces, and a significant effect of mean wing luminance. There was no evidence that false depth cues provided the same protective benefits as physical surface disruption in three-dimensional prey, perhaps because our treatments did not mimic the complexity of patterns found in natural animal markings. Our findings indicate that disruption of surface continuity is an important strategy for concealing a three-dimensional body shape.

## 1. Introduction

Relentless selection pressure from predators has produced a variety of strategies for visual camouflage. One of the most common and effective visual camouflage techniques is disruptive coloration (e.g. [1–6]). Camouflage using disruptive coloration can occur when an animal's markings present high contrast, false edges that intersect the edge of the body and distract from the natural edges, and/or provide false depth cues that break the body surface into apparent multiple depth planes [2–10]. This latter effect may be particularly effective if the edges of colour patches are accentuated (edge enhancement) so that light patches have lighter edges, and dark patches have even darker edges, as is commonly observed in reptiles, amphibians and Lepidoptera [1,11,12]. Such patterns are thought to enhance camouflage by hijacking the viewer's mechanisms of depth perception [13,14].

There are several ways in which enhanced edges may interfere with visual processing. Osario & Srinivasan [14] modelled the receptive fields of edge detectors, demonstrating that enhanced edges over-excite the edge detectors, which may cause the segregation of the pattern edges, rather than the animal's real boundaries. In addition, false edges may interfere with higher levels of processing if they provide false cues to object depth. Using humans as predators, Egan *et al*. [11] found that camouflaged, snake-shaped targets were harder to detect when they had enhanced edges, and that these targets were judged by viewers to have more depth information than control targets without enhanced edges. A subsequent study also found that prey with enhanced edges were harder to classify (i.e. recognize as discrete prey types) than those without enhanced edges [12]. Both studies suggest that enhanced edges can interfere with depth perception in humans, causing adjacent patches of colour to be grouped separately, rather than being recognized as belonging to the same object [11]. Currently, the significance of enhanced edges has only been tested using edge detection models [14] and human vision [11,12,15] and it is not clear whether these patterns confer an increase in survival probability when viewed by non-human visual predators.

If enhanced edges enhance camouflage by apparently disrupting the continuity of the body's surface, then prey with physically disrupted body surfaces should be harder to detect and/or recognize than prey with continuous surfaces. While a previous study found that ‘false holes' in butterfly wings confer a survival advantage to prey [16], this work did not explore whether similar benefits could be obtained using hole-like markings with enhanced edges. Here, we determine whether (1) physical surface disruption can provide a survival advantage to three-dimensional prey, and (2) whether enhanced edge markings confer a survival advantage over disruptive markings. These questions were addressed using a predation experiment in which three-dimensional-printed, moth-like targets were presented to wild avian predators [4,7]. To investigate the possible mechanisms for differential survival of the target types, we used digital photography and avian visual modelling to evaluate the camouflage metrics of the targets *in situ.*

## 2. Material and methods

### (a) Target creation

**Figure 1.**
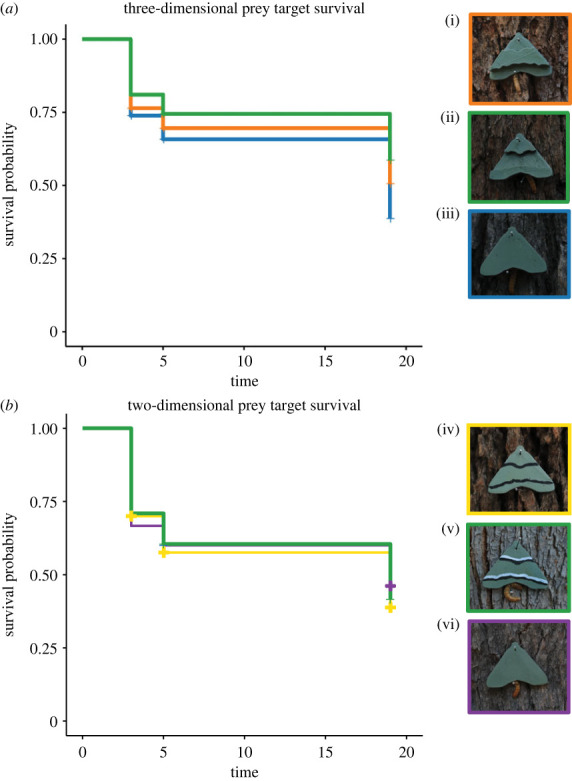
Changes in survival probability over time (in hours) for three-dimensional prey (*a*; i:iii) and two-dimensional prey treatments (*b*; iv:vi). Treatments with disrupted surfaces (i and ii: orange and green lines) had higher survival than three-dimensional targets with continuous surfaces (iii: blue line) (*a*). There was no difference in survival between the two-dimensional treatments (*b*; iv: yellow line (disruptive pattern), v: green line (enhanced edges), vi: purple line (no pattern). Images (i)–(vi) show targets photographed in field, illustrating the range of illumination conditions and backgrounds.

Prey targets were generated using Fusion 360 (Autodesk, Inc., California, USA) three-dimensional modelling software. Target shape was based on a common disruptive pattern seen in Lepidoptera in the area (see electronic supplementary material A). We produced 30 replicates for each of six different target designs (wing diameter: 40 mm). Three target designs were ‘three-dimensional' targets (maximum thickness = 5 mm) and three were ‘two-dimensional' targets (thickness = 1 mm). The two-dimensional targets all had continuous (flat) surfaces. Our use of terminology (two-dimensional and three dimensional) is to help distinguish the concepts and is not based on the classification or perception of these shapes by avian predators. Of the three-dimensional targets, two had disrupted surfaces, with either the central (figure 1*a*(i)) or peripheral parts raised (figure 1*a*(ii)), while one had a continuous surface (figure 1*a*(iii)). The two versions of the disrupted surface targets represent two perceptual outcomes that could result if the two-dimensional patterns (disruptive and enhanced edge; see below) generate false ‘pictorial' depth cues [1,13]. Due to time constraints, it was not possible to add continuous three-dimensional models with disruptive and enhanced edge patterns to the experimental design. All targets had a small pinhole at the top to aid fixation of the targets to trees. Targets were printed with polylactic acid (PLA) using Ultimaker Cura slicing software and a Creality 3D printer.

To ensure that the colour of the targets provided a good match to the visual background (tree bark), we used digital photography to measure the bark colour of local marri trees (*Corymbia calophylla*) (see electronic supporting material, B). Bark images (*n* = 10 trees) were linearized and modelled for avian vision using chart-based cone catch modelling in Mica Toolbox [17] for ImageJ software [18]. We used the blue tit (*Cyanistes caeruleus*) because it is insectivorous and because the spectral sensitivities of local birds are not known. Sample targets were painted and photographed *in situ* for subsequent measurement and identification of the closest paint match (British Paints™: Pale Eucalypt; A3944). Targets were then sprayed, and additional paint applied to two of the two-dimensional target designs; disruptive markings were created for one set of targets using black paint (figure 1*a*(iv)), while markings with enhanced edges were created for another set of targets (figure 1*a*(v)) using black and white paint. Targets with two-dimensional continuous surfaces were non-patterned (figure 1*a*(vi)). Markings were hand-painted using a stencil and all targets were sprayed with water repellent spray (Boyle Industries, Australia; matt finishing sealer). We anticipated that the application of water repellent spray would mask any difference in odour caused by the application of the black and white paint.

### (b) Field trials

Field trials were conducted in April–May 2021 in Balingup Forest, Western Australia (S 33 46.160, E 116.04.144), which is inhabited by a variety of insectivorous birds including scarlet robins (*Petroica boodang*) and rufous treecreepers (*Climacteris rufus*). We performed nine blocks (replicates) of the experiment, with each block being conducted in a different part of the forest (>500 m apart). Each block consisted of 10 targets per treatment giving 60 targets per block.

Each block consisted of a transect (approx. 1 km long) of marri trees at least 3m apart. Targets were pinned in a vertical orientation (approx. 1.5 m above ground) with treatment randomized. A food source for the birds was provided by pinning a live mealworm (*Tenebrio molitor*) under the base of each target, leaving part (approx. 1.7 cm) of the mealworm exposed. For each block, targets were set out between 09.00 and 11.00 h and checked for predation after 3, 5 and 19 h. At each check, all targets were inspected for signs of predation by birds (mealworm missing), spiders (mealworm exoskeleton present) or ants (ants still visible near target). Targets that had undergone predation were photographed *in situ* (using a Canon EOS 80D DSLR; for subsequent modelling of luminance information; see Image analysis) and removed, with all remaining targets being removed at the final check. To obtain a random set of images for the camouflage metrics, we numbered the targets in each treatment and photographed all those that were predated, as well as those with an even number that survived. We did not photograph all of the targets because processing the images is very time consuming. Images were captured approximately 50 cm from each tree and included a greyscale for subsequent linearization and visual modelling.

### (c) Statistical analysis

Data were analysed using mixed effects Cox regression (coxme) models [19] in R [20]. Targets were considered censored if there were signs of attack by predators other than birds and in instances where mealworms were still present at the last check. Survival time (SVT) in hours was the time between when the targets were placed and when the mealworm was taken by a bird and corresponded with the target check times (3, 5 and 19 h).

The response variable was generated using the ‘survival' package [21], entering survival time (in hours) and whether the subject was censored (=0) or taken by a bird predator (=1). Treatment was entered as a fixed effect and block (nine levels) as a random effect. We chose not to compare all possible contrasts between the six treatments, as this would result in a lot of comparisons, some of which would not be biologically relevant. We therefore conducted planned comparisons *a priori* and adjusted the *p*-values to control the false discovery rate [22]. We first compared the survival probability of three-dimensional targets with continuous surfaces and disrupted surfaces (*n* = 267). We then compared the survival of two-dimensional targets with no patterning to two-dimensional targets with disruptive and enhanced edge patterns (*n* = 267). We also investigated whether survival depended on whether the shape was three-dimensional or two-dimensional. The significance of the fixed effects was tested against each final model using log-likelihood ratio tests (LRT).

### (d) Image analysis

We used a subset of the data (i.e. all survival data that had an associated image) to examine the effect of camouflage metrics on survival probability. The images were captured at the same time as the target checks and included predation events and targets that survived at each check. We used the polygon tool in ImageJ [18] to mark the region of interest (ROI: the target) before images were linearized and converted to the cone catch model using Mica Toolbox [17]. The ROIs in these images were used to measure the mean luminance of the target; we focused on birds' double cones (luminance information) as luminance is considered important for visual detection in birds [23]. Mean wing luminance was standardized (each value divided by the mean) and input as a covariate in the models.

## 3. Results

**Figure 2.**
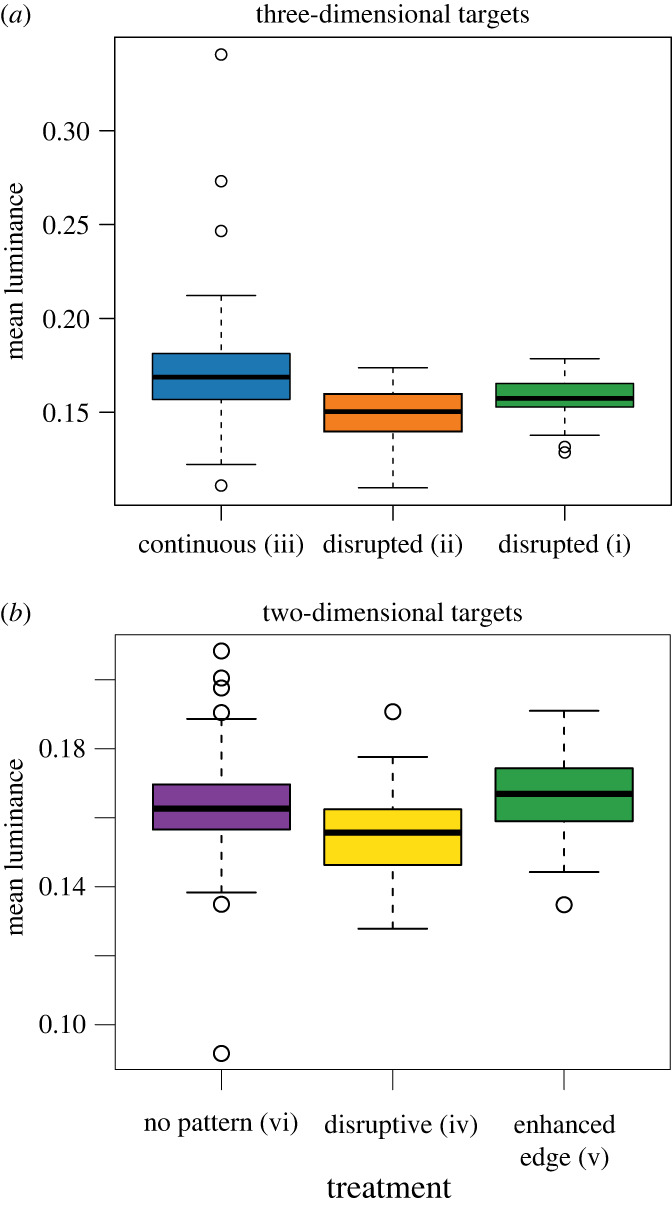
Differences in mean luminance (double cones for blue tit vision) for three-dimensional targets with continuous (iii) and physically disrupted surfaces (i and ii; *a*). Targets significantly differed in mean luminance (ANOVA: *F*_2,186_ = 17.25, *p* < 0.001); targets with disrupted surfaces had lower luminance than those with continuous surfaces ((i) versus (iii): *p* < 0.001; (ii) versus (iii); *p* = 0.002), but there was no difference in luminance between the two disrupted surface types ((i) and (ii); *p* = 0.08). Differences in luminance for two-dimensional targets with no patterning, disruptive patterning, and enhanced edges are shown (*b*). Targets without patterning and targets with enhanced edges had higher mean luminance than targets with disruptive patterns, but there was no difference in luminance between non-patterned targets and those with disruptive patterns (Tukey tests; *p* < 0.05).

We found an overall effect of treatment on target survival time (*χ*^2^ = 11.88, d.f. = 5, *p* = 0.036); three-dimensional targets with disrupted surface type (ii) had 59% the risk (*β* = −0.53) of the equivalent three-dimensional targets with continuous surfaces (target type iii; *p*_adjust_ = 0.039; figure 1*a*). There was no difference in survival between physically disrupted surface type (i) and either continuous surface targets (iii; *p*_adjust_ = 0.248) or disrupted surface targets (ii) (*p*_adjust_ = 0.248). Incorporating the image metrics, the full model included significant effects of both treatment (*χ*^2^ = 11.75, d.f. = 5, *p* = 0.038) and mean wing luminance (*χ*^2^ = 6.77, d.f. = 1, *p* = 0.009). However, after correcting for the false discovery rate, only luminance remained significant (*p*_adjust_ = 0.04; *β* = −1.67); targets with low luminance had 19% the risk of equivalent targets with higher levels of luminance (figure 2*a*).

For the flat two-dimensional targets, there was no difference in survival between unpatterned targets (vi) and those with disruptive patterns (iv; *p*_adjust__=_ 0.790) or enhanced edges (figure 1*b*; *p*_adjust__=_ 0.790). There was also no difference in survival between targets with disruptive or enhanced edge patterns (*p*_adjust_ = 0.790), despite these targets varying in luminance (*F*_2,193_ = 14.8, *p* < 0.001; figure 2*b*). Finally, there was a significant difference in survival between three-dimensional prey and two-dimensional prey (*χ*^2^ = 5.07, d.f. = 1, *p* = 0.024); two-dimensional targets had lower survival probability than three-dimensional targets (*β* = 0.27, z = 2.25, *p* = 0.025) (see electronic supplementary material, C).

## 4. Discussion

We investigated how depth cues might influence animal camouflage by investigating the survival benefits of surface disruption for three-dimensional prey, and the advantages of disruptive coloration and enhanced edge markings for two-dimensional prey. We found that three-dimensional prey with a physically disrupted surface had a survival advantage over three-dimensional prey with continuous surfaces. This suggests that physical surface disruption is a successful camouflage strategy for three-dimensional prey. While we anticipated that enhanced edges in two-dimensional prey might have the same effect (based on studies with human observers [11,12,15]), we found no evidence for increased survival of two-dimensional prey with enhanced edges or disruptive markings. These findings suggest that the two-dimensional markings did not provide the intended (false) depth/disruptive cues, and/or that birds were employing other visual processing mechanisms to break camouflage.

There are several explanations for the survival benefits of physical surface disruption, although our experimental design does not allow us to establish the underlying mechanisms. The benefits of physical surface disruption could be explained by perceptual processing by avian predators. The changes in depth across the targets' three-dimensional surface may have generated strong cues for perceptual segregation, promoting incorrect boundary resolution and causing the target to be misidentified [13]. To demonstrate a role for perceptual segregation, we would need to show that birds perceived the targets as separate objects of varying surface depth rather than as single, whole objects. A study of the protective benefits of ‘false holes' found that butterfly like prey with false holes had similar survival to prey with real holes, with both having a survival advantage over prey with intact wing surfaces [16]. Although surface disruption provides a good explanation for these findings, the role of depth perception remains unclear [16]. Nevertheless, Costello *et al.* [16] were able to demonstrate similar findings (i.e. a survival benefit associated with real holes and false holes) when translating their experiments to search tasks with human observers. Another possible explanation for our findings is neophobia in the birds' foraging behaviour [24]; birds may have avoided prey with disrupted surfaces because they are novel relative to prey with continuous surfaces. If this is the case, neophobia could provide additional survival benefits for two-dimensional patterns that produce false depth cues, but this possibility requires investigation of bird perception and behavioural responses to novel prey types.

Our finding of a significant effect of mean wing luminance on survival time suggests that background matching of three-dimensional visual information may play an important role. For example, a relatively large surface with approximately constant luminance (i.e. a flat, continuous surface) may be highly conspicuous when viewed against a (textured) background with variable surface depth (i.e. bark). Thus, some of the benefits of surface disruption might be due to background matching whereby the prey's visual signature matches the visual cues in the background that are associated with changes in surface depth [13]. Prey orientation relative to the surface texture can also play an important part in camouflage [25]. For example, the geometrid moth *Jankowskia fuscaria* uses tactile cues to detect the physical structure of the background to select a resting position that optimizes camouflage [26]. In the present study, there would have been a mismatch between the vertical furrows of the bark background and the horizontal changes in surface depth in the three-dimensional disrupted surface targets. Nevertheless, for backgrounds with strong depth cues, it may be better to have a poorly aligned, disrupted surface than a continuous surface, where conspicuousness may be high irrespective of orientation.

Our analysis of the two-dimensional prey targets found that enhanced edge markings provided no survival advantage over targets with disruptive markings or non-patterned controls. Our simple patterns were hand painted and may not have provided sufficient visual information to fool the predators into breaking up the body outline (disruptive patterns) or breaking up the body surface (enhanced edges). Indeed, previous studies with human observers have been based on natural animal markings [11,12], which feature strong changes in luminance (e.g. a ‘step' component [14]) when measured across a transect perpendicular to the patterning. Indeed, Egan *et al*. [11] specifically examined whether the shape of the step gradient was important for camouflage, finding that when no gradient was present (adjacent dark and light bands, as in our study), targets were no better camouflaged than controls. Although their study [11] found that edge enhancement enhanced crypsis, detection times were dependent on the shape of the step gradient and the presence of shadows in the background image. A key challenge for future research is to understand the perceptual effects generated by enhanced edges: do they facilitate image segregation to break surface continuity and/or do they mimic the visual properties of natural textures to enhance background matching?

## Data Availability

Supporting data and code are available from the Dryad Digital Repository: https://doi.org/10.5061/dryad.7wm37pvxr [27]. The data are provided in the electronic supplementary material [28].
